# Clinical Efficacy of Platelet Derivatives in Periodontal Tissue Regeneration: An Umbrella Review

**DOI:** 10.1155/2023/1099013

**Published:** 2023-07-03

**Authors:** Carlos M. Ardila, María Pertuz, Annie Marcela Vivares-Builes

**Affiliations:** ^1^Biomedical Stomatology Research Group, Universidad de Antioquia UdeA, Medellín, Colombia; ^2^Fundación Universitaria Visión de Las Américas, Medellín, Colombia

## Abstract

**Objectives:**

This umbrella review aimed to consolidate the best available evidence regarding the clinical efficacy of platelet derivatives in the treatment of periodontal defects associated with periodontitis and in the management of mucogingival deformities.

**Materials and Methods:**

The “umbrella review” strategy was used to identify systematic reviews and meta-analyses. The search was performed without language restrictions and updated at the end of February 2023. The PubMed and Scopus databases, together with gray literature, were included in the search.

**Results:**

The search yielded 412 studies. Subsequently, 12 articles were selected for further examination based on relevance. Finally, eight systematic reviews and meta-analyses were assessed. Regarding intrabony defects, in terms of clinical attachment level (CAL) gain, platelet-rich fibrin (PRF) was observed to lead to a statistically significant attachment gain compared to surgical therapy alone. PRF was also found to show greater CAL gain compared to platelet-rich plasma (PRP) and other biomaterials. The parameter probing depth decreased significantly when PRF was used compared to surgical therapy alone (*P* < 0.05). Similar results were seen when leukocyte- and platelet-rich fibrin (L-PRF) was applied. In terms of radiographic bone fill, both PRF and PRP showed significantly greater bone fill compared to surgical therapy. Regarding the results of periodontal plastic surgery, PRF favored a slight root coverage compared to the coronally displaced flap. This result depended on the number of PRF and L-PRF membranes used, but Emdogain or connective tissue graft produced better results regardless. However, an improvement in the healing of periodontal tissues was reported.

**Conclusions:**

Therapies with platelet derivatives applied to intrabony defects provided superior regenerative results compared to monotherapies, except in the case of root coverage.

## 1. Introduction

Periodontal health implies the absence of inflammation associated with gingivitis, periodontitis, and mucogingival deformities, such as gingival recessions, considering individual anatomical, and morphological variability [1]. Periodontitis is an inflammatory disease of a multifactorial nature that affects the supporting tissues of the teeth. Here, the interaction between factors such as the immune response, lifestyle, habits, parafunctions, anatomical conditions, and occlusal trauma [2, 3] associated with dysbiotic plaque biofilms leads to the progressive destruction of tooth support tissues, generating residual soft, bone, and intraosseous tissue defects of varying degrees, up to the consequent dental loss [3, 4]. Periodontitis is a prevalent disease that affects more than 40% of individuals in the United States. The severe form of the disease has a global frequency of 11% [2].

Gingival recession is defined as the apical displacement of the gingival margin with respect to the cementoamelic junction associated with the loss of attachment and exposure of the root surface [5]. There is still insufficient scientific evidence for the associated etiological factors, which are mainly based on clinical observations and include a thin periodontal biotype, absence of attached or attached gingiva, reduction in the thickness of the alveolar bone due to the inadequate position of the tooth within the alveolar process, orthodontic movements, and nonoptimal restorative procedures and oral hygiene [5, 6]. This mucogingival condition causes deterioration of the exposed root surfaces, development of carious and noncarious cervical lesions, dentinal hypersensitivity, loss of dental attachment, predisposition to localized inflammatory processes, and deterioration in dentogingival esthetics [6]. Treatment is indicated when it is difficult to control biofilm formation and when restorative or orthodontic treatment is required depending on the direction and size of the movements [7]. It is important to note that gingival recessions and a lack of keratinized tissue are highly prevalent in the adult population [5].

The clinical manifestations of gingival recessions and the residual bone defects, sequelae of periodontitis, are as variable and diverse as the patients treated. Therefore, the therapeutic approaches must be individualized and include surgical therapies with a regenerative approach to reduce morbidity and improve results in terms of insertion level gains, thereby increasing the probability of dental survival; this currently represents a surgical challenge due to the innovations in the techniques and biomaterials used [8, 9].

Platelet derivatives are autologous biomaterials that have been introduced in recent years as a scaffold to allow better healing and in regenerative treatments of periodontal diseases and mucogingival deformities [10]. They are associated with the presence of growth factors such as transforming *β*1, platelet-derived growth factor (PDGF), vascular endothelial growth factor, glycoproteins such as thrombospondin-1, and cytokines such as interleukins (IL)-1*β*, IL-4, and IL-6 [11, 12], in addition to fibrin, fibronectin, and vitronectin, which act as connective tissue matrix and adhesion molecules for more efficient cell migration [13–15].

In dental procedures, this protocol was adopted in 2001, and in 2009, Dohan Ehrenfest et al. [16] established a classification for the different platelet concentrates. Thus, according to their leucocyte and fibrin content, platelet-rich products are currently classified as follows: (1) PRP: (a) pure platelet-rich plasma (P-PRP) and (b) leukocyte- and platelet-rich plasma (L-PRP); (2) platelet-rich fibrin (PRF): (a) pure platelet-rich fibrin (P-PRF), (b) L-PRF, and (c) injectable platelet-rich fibrin (I-PRF).

Although different systematic reviews and meta-analyses have studied the use of platelet derivatives for the management of intrabony defects [17–23] and mucogingival deformities [19, 24], most of them present results only on PRF [17, 18, 20–22, 24]. Furthermore, all these reviews present only encouraging results in terms of healing and postoperative morbidity, except for reviews by Miron et al. [18] and Najeeb et al. [22] that present clinical results comparable with those of open flap debridement plus bone graft. On the other hand, Tavelli et al. [23] found that the type of bone graft material and biological agent used had a substantial impact on the clinical and radiographic results of intrabony defect treatment. These reviews also indicate a lack of evidence and the lack of a convincing role for the use of PRF in the management of intrabony defects and gingival recessions. Therefore, the objective of this umbrella review was to consolidate the best available evidence considering the clinical efficacy of platelet derivatives in the treatment of periodontal defects associated with periodontitis and in the management of mucogingival deformities.

## 2. Materials and Methods

A detailed protocol was developed and registered in PROSPERO (International Prospective Register of Systematic Reviews-404600). This umbrella review was developed following the recommendations of the Preferred Reporting Items for Systematic Reviews and Meta-Analyses (PRISMA) statement [25]. The “umbrella review” strategy was used to identify systematic reviews and meta-analyses [26] aimed at evaluating the clinical efficacy of platelet derivatives in the treatment of periodontal defects associated with periodontitis and in the management of mucogingival deformities.

### 2.1. Search Strategy

The initial search was performed by two authors (AV and MP), independently and without language restrictions, and updated at the end of February 2023. The PubMed and Scopus databases, together with gray literature, were used to identify titles and abstracts related to the objective of this review. The search strategy was adapted to the databases used, and the following MeSH terms and Boolean operators were used: “Bone Regenerations” OR “Regeneration,” “Bone Dentistry” AND “Plasma,” “Platelet-Rich” OR “Platelet Rich Plasma” OR “Fibrin,” “Platelet-Rich” OR “Platelet Rich Fibrin” OR “L-PRF” OR “Leukocyte” AND “Platelet-Rich Fibrin” AND “Bone Regenerations” OR “guided tissue regeneration” OR “guided tissue repair,” “systematic” AND “metanalysis” AND “review” AND “bone” AND “regenerations” OR “regeneration,” AND “bone” AND “dentistry” AND “plasma,” AND “platelet rich OR “platelet” AND “rich” AND “plasma” OR “fibrin,” AND “platelet-rich” OR “platelet” AND “rich” AND “fibrin” OR “l-PRF” OR “leukocyte” AND “platelet rich” AND “fibrin” AND “bone” AND “regenerations” OR “guided” AND “tissue” AND “regeneration” OR “guided” AND “tissue” AND “repair.”

### 2.2. Question

The PICO question was defined as follows: In patients with intrabony defects or mucogingival deformities, what is the clinical efficacy of platelet derivatives (PRF, L-PRF, and PRP) alone or combined with other biomaterials in terms of gain in clinical attachment level (CAL), decreased probing depth (PD), and percentage of root coverage, plus radiographic bone fill of intrabony defects, gain of attached gingiva, and increased width of keratinized gingiva in the case of mucogingival deformities?

### 2.3. Inclusion Criteria

This umbrella review aimed to identify systematic reviews and meta-analyses conducted in humans and derived from clinical trials according to the recommendations of the Cochrane Collaboration Group. Reviews that included clinical trials of periodontal regenerative surgical therapy in adult patients presenting intrabony defects or gingival recessions using platelet derivatives (PRF, L-PRF, and PRP) alone or combined with other biomaterials, with a minimum follow-up of 6 months, were considered eligible.

### 2.4. Exclusion Criteria

Systematic reviews and meta-analyses that included animal studies, studies derived from theoretical reviews, or critical and theoretical essays, and those that included regenerative procedures for conditions other than intrabony defects and procedures other than root coverage in the management of mucogingival deformities, were not considered.

### 2.5. Outcome Variables

The main outcome variables included CAL and decrease in PD in intrabony defects and the percentage of root coverage in mucogingival deformities after periodontal plastic surgery procedures.

The secondary outcome variables included radiographic bone fill in intrabony defects and gain of attached gingiva and increased width of keratinized gingiva in mucogingival deformities after periodontal plastic surgery procedures.

### 2.6. Review Process

Two researchers (AV and MP) reviewed the titles and abstracts and selected systematic reviews and meta-analyses to evaluate the full text for suitability. Any disagreements that arose between them were discussed with the third author (CA). The Kappa test was utilized to measure the level of agreement between observers (*K* = 96).

### 2.7. Data Collection

A tool was proposed to include the most pertinent information from the selected reviews. This tool was implemented individually by each of the investigators. Successively, the records were contrasted. Verified information included the authors' names, date of publication, intervention and control, and comparison between the groups (main and secondary outcome variables).

### 2.8. Critical Appraisal of Selected Reviews

The methodological quality of the included articles was evaluated using the PRISMA checklist for systematic reviews [25]. Each item received a score of 1 if it met the specific criteria and a score of 0 if the criteria were not met, unclear, or not applicable. An overall score related to the quality of the review (sum of individual item scores) was then calculated. The Amstar-2 guide was used to establish the degree of confidence in the review and the risks of bias [27]. These evaluations were carried out independently by two authors (AV and MP) and were analyzed and reviewed by the third author (CA).

## 3. Results

### 3.1. Study Selection

**Figure 1:**
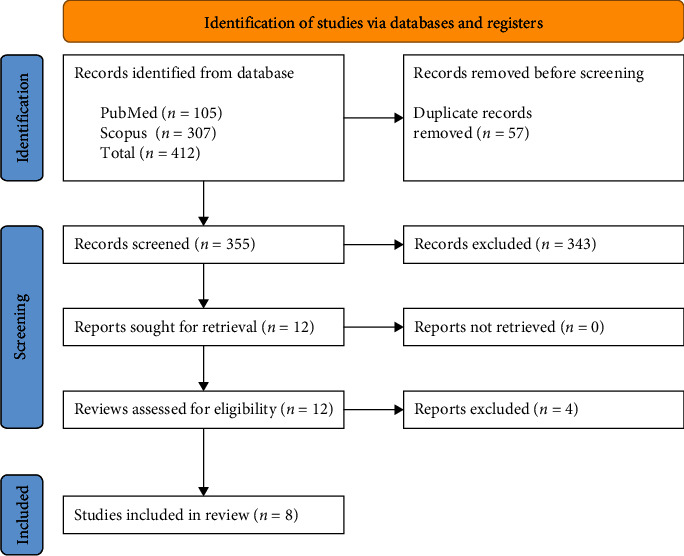
Flowchart of the review's selection method.

The bibliographic search yielded 412 studies. Subsequently, 12 articles were considered relevant and selected for further examination. Four articles were excluded because they included procedures outside the scope of the present review, such as maxillary sinus floor lift procedures, regeneration with dental implants, and alveolar preservation. Finally, eight systematic reviews and meta-analyses [10–17] that met all the inclusion criteria were assessed. The complete data extraction process is explained in Figure 1.

### 3.2. Characteristics of Included Studies

Although the results could be assessed quantitatively with existing meta-analyses, it was decided to also include systematic reviews to capture the greatest amount of evidence by identifying the largest possible number of available primary studies and thus better reflect current evidence on the topic under study in this review. The eight included reviews were published between 2016 and 2022. Six of the studies also performed a meta-analysis (Table 1).

Seventy-five primary studies were included in the systematic reviews and meta-analyses, of which 60 determined the clinical efficacy of platelet derivatives. The types of platelet concentrates evaluated in this review were PRP, PRF, and L-PRF. The studies selected compared surgical therapy alone and supplemented with PRP, PRF, and L-PRF; in addition, they compared the use of PRF and PRP. They also compared the clinical efficacy of these derivatives alone and with the addition of various biomaterials such as bone substitutes and barrier membranes with guided tissue regeneration techniques (Table 2).

### 3.3. Synthesis of the Evidence

Regarding intrabony defects, Table 1 shows that in terms of CAL gain, PRF led to a statistically significant attachment gain compared to surgical therapy alone [10, 11, 13, 14, 17]. When comparing PRF with PRP and other biomaterials, PRF was found to show greater CAL gain than the PRP and amniotic membrane subgroups [14]. Furthermore, PRF was found to show statistically significant differences in CAL compared to demineralized freeze-dried bone allograft (DFDBA) alone [14]. On the other hand, when contrasting the use of PRF with Emdogain (EMD), no statistically significant differences were observed in CAL [17].

Considering the choice of platelet derivatives and other biologic agents, all groups revealed improvement in CAL gain results. Any biologic agent improved CAL compared with surgical therapy alone, including allografts (−0.45; 95% CI: −0.89, −0.01), xenografts (−0.41; 95% CI: −0.77, −0.04), EMD (−0 0.61; 95% CI: −0.81, −0.38), and absorbable barrier membranes (−0.79; 95% CI: −1, −0.41), or platelet derivatives such as PRP (−0.58; 95% CI: −0.91, −0.26) and PRF (−0.82; 95% CI: −1.08, −0.56). However, it was indicated that in the case of biomaterials, it would not be beneficial to use barrier membranes because they nullify the overall effect of the biomaterial [23].

In terms of PD, the use of PRF decreased this parameter significantly (*P* < 0.05) compared to surgical therapy alone [17, 20]. Similar results were seen when L-PRF was utilized (mean difference: 1.1 mm; *P* < 0.001; 95% CI: 0.6–1.6) [19]. When comparing PRP and PRF, better results were found with PRF; furthermore, PRP and PRF showed statistically significant differences in PD reduction (mean deviation: 0.88; 95% CI: 0.41–1.34) compared to DFDBA alone (mean deviation: 0.47; 95% CI: 0.14–0.80) [21]. Another meta-analysis reported a decrease in PD with the use of PRP (−0.41; 95% CI: −0.66, −0.16), PRF (−0.57; 95% CI: −0.76, −0.38), and EMD (−0.55; 95% CI: −0.71, −0.39) [23]. On the other hand, no statistically significant differences were found in terms of PD when using PRP together with bone grafting compared to guided tissue regeneration [20].

Table 1 also shows that in terms of radiographic bone fill, both PRF and PRP showed significantly greater bone fill compared to surgical therapy [18–20]. Considering the biological agents in terms of bone refilling, all groups showed significant improvements with PRP, PRF, and EMD. However, the differences between EMD and PRF were statistically significant in favor of PRF (9.89; 95% CI: 1.04, 18.75) [23].

Regarding the results of periodontal plastic surgery (Table 1), PRF favored a slight root coverage compared to the coronally displaced flap (CAF); however, this result depended on the number of PRF and L-PRF membranes used, indicating that a minimum of three membranes are required to optimize results, although this does not lead to better results compared to EMD or connective tissue graft (CTG) [17]. The greatest gain of keratinized gingiva was achieved with CAF in combination with CTG, compared to PRF [17]. When comparing CAF + L-PRF versus CAF alone, there was a trend toward improvement from L-PRF; however, the results were not statistically significant regarding the gain in keratinized gingiva width, tissue thickness, and root coverage. Statistically significant differences were only found for the reduction in recession depth at 6 months using L-PRF (mean difference: 0.6 mm; *P* < 0.01; CI: 0.2–1.1). When evaluating the effect of CAF + L-PRF versus CAF + CTG, no statistically significant differences were found in the gain of keratinized gingiva and reduction of recession [19]; however, another review indicated that the increase in the extent of keratinized gingiva was significantly greater when using CTG compared with PRF for studies of a duration greater than or equal to 6 months [24]. It was then concluded that the additional use of PRF for the treatment of gingival recessions did not lead to any additional benefit in root coverage (*P* = 0.57). The use of PRF does not improve root coverage or the width of keratinized gingiva in Miller classes I and II gingival recessions compared to other treatment modalities, including EMD or CTG [24]. However, an improvement in the healing of periodontal tissues was reported [17, 19, 24].

### 3.4. Risk of Bias

Only two reviews had a moderate risk of bias, while the other six had a low risk according to the PRISMA scale. However, the Amstar-2 guide showed a low level of confidence in most of the studies (Table 3), which indicates that these systematic reviews have serious flaws and could not supply a precise and comprehensive summary of the available investigations that study the question of interest. Moreover, the clinical trials included in these reviews presented great heterogeneity in their designs, reflected in the use of different clinical procedures, comparison groups, and outcome variables, among others, an issue that made it difficult to perform adequate meta-analyses.

## 4. Discussion

This umbrella review sought to consolidate the evidence available in systematic reviews and meta-analyses based on clinical studies, mostly randomized controlled clinical trials.

This review highlights the abundant scientific evidence demonstrating a reduction in PD, CAL gain, and radiographic bone filling in intrabony defects, as well as an improvement in postoperative healing parameters, in periodontal plastic surgery using platelet derivatives. These results justify the clinical application of platelet derivatives due to their efficacy as well as the economic implications of their lower cost and possibly greater clinical acceptance by patients since these products are autologous biological derivatives. Generally, regarding intrabony defects, the results are better if platelet derivatives are added to bone regeneration materials that include bone substitutes (autogenous, allogeneic, xenograft, and even alloplastic), with statistically significant improvements compared to procedures without these derivatives.

PRF produced superior regenerative results to PRP in terms of improvement in CAL and PD. The studies by Tavelli et al. [23] and Zhou et al. [21] suggested that one of the main advantages of PRF is the formation of a fibrin-dense clot that contributes to the prolonged release of growth factors over time, leading to its exerting the most significant adjuvant effect in soft tissue healing. On the other hand, PRP has a unique impact on hard tissue reconstruction in the treatment of periodontal intrabony defects combined with bone substitutes, whereas EMD demonstrates little additional benefit. These results can be explained by the biochemical characteristics of platelets that contain biologically active proteins, including PDGF and transforming growth factor *β*, that promote protein synthesis in bone tissues and have a chemotactic effect on osteoblastic and endothelial cells, as well as inhibitory effects on osteoclasts. Platelets also contain insulin-like growth factor-I (IGF-I), which stimulates osteoblast proliferation and increases osteocalcin expression for extracellular matrix synthesis. Furthermore, the combination of IGF-1 and PDGF promotes the speed and quality of wound healing. All these proteins, in turn, bind to a developing fibrin mesh of the extracellular matrix, generating a chemotactic gradient for cell recruitment. L-PRF, for its part, has the additional benefit of including leukocytes that favor microbial inhibition and potentiate the effect of cell differentiation and regeneration in tissues [11, 12, 17].

Although leukocyte and platelet cytokines play an important role in the healing abilities of PRF and L-PRF, the fibrin matrix that supports these elements is suggested to be responsible for their therapeutic potential. The keys to tissue regeneration lie in its angiogenic potential, control of the immune system, potential to recruit circulating stem cells, and ability to ensure wound closure/healing without alterations by epithelial tissues [18, 19, 23].

It is important to highlight that although there is sufficient biochemical clarity that explains and justifies the use of these platelet derivatives, true histological periodontal regeneration in humans has not yet been demonstrated; therefore, more studies are required to characterize the physical properties of these platelet derivatives, especially since they have been indicated to prevent the apical migration of the epithelium in periodontal defect treatment and other regenerative procedures [14]. Dohan et al. [100] suggested that PRF and L-PRF are not only platelet concentrates but also immunological nodules capable of stimulating defense mechanisms. Furthermore, the important inflammatory regulation observed in treated surgical sites is likely the result of the effects of cytokines trapped in the fibrin network that is released during remodeling.

PRF has been widely used as a bioactive matrix in numerous studies for the root coverage of gingival recessions; however, its clinical efficacy is limited [17, 24]. Its use does not lead to an improvement in clinical variables such as the increase in the width of the keratinized gingiva and the percentage of root coverage; nevertheless, some studies have reported that it can be used in periodontal plastic surgery as an adjunct for pain management, lower morbidity, and faster wound healing, especially in donor beds such as the palate area since it significantly accelerates wound healing and reduces patient morbidity. However, it is important to highlight that regarding these variables of early wound healing, greater standardization of the studies' measurement methods is required, for example, through the use of the cure index introduced by Wachtel et al. [17, 24, 101].

In terms of radiographic bone fill, results were conflicting regarding the benefit of PRP in combination with bone graft materials compared to the use of graft materials alone. The systematic review by Panda et al. [20] did not report statistically significant differences while other reviews indicated an additional benefit with the use of this platelet derivative [18, 19, 23]. In the systematic review by Najeeb et al. [22], no significant differences were observed between PRF and PRP either. Furthermore, no differences in PD and CAL were observed when PRF and EMD were compared. However, it is important to highlight that this review did not include a meta-analysis, and its findings contrast with those of other reviews that performed a quantitative analysis and had a low risk of bias [18, 19, 23].

Tavelli et al. [23] stated that the benefit of using a barrier membrane would be negated by the presence of biologics including PRP, PRF, and L-PRF. Moreover, PRP and PRF did not show any additive beneficial effects when combined with bone graft and membrane for the treatment of intrabony defects. This aspect must be considered in terms of cost–benefit while planning clinical procedures [20, 23].

This umbrella review allows for greater clarity regarding the use of platelet derivatives in periodontal and mucogingival regeneration therapies since it brings together the best available evidence, allowing many questions and doubts that arise in clinical practice to be answered. However, the highly heterogeneous analyses of the included reviews may increase bias in the individual and overall results of this umbrella review.

## 5. Conclusions

1. Therapies combined with platelet derivatives (PRP, PRF, or L-PRF) produce superior regenerative results in intrabony defects in terms of improvement of CAL, PD, and bone filling, compared to monotherapies and surgical periodontal therapeutic procedures alone.
2. The use of PRP in surgical therapies of intraosseous defects provides more benefits in terms of radiographic bone filling and clinical attachment gain when combined with bone substitutes, being the most recommended xenograft in intraosseous defects.
3. PRF and L-PRF can be used alone in surgical therapies of intrabony defects with improved results in terms of improvement of CAL, PD, and bone filling, compared to the use of PRP without bone substitute or only surgical therapy.
4. In GRT therapies, the type of defect and the surgical technique to be used should be diagnosed appropriately if the defect is not contained. If extensive regeneration of a defect wall is required, it is recommended to use a barrier membrane and bone substitute, which, in turn, can be combined with PRP. However, it is not recommended to use PRF and L-PRF and other biologics because they do not confer an additional benefit to that already provided by the barrier membrane.
5. In periodontal plastic surgery where the main objective is to achieve not only root coverage but also an increase in the band of keratinized gingiva, it is not recommended to replace the autologous CTG or xenograft with PRF or L-PRF, since they do not provide significant advantages regarding clinical results (PD, CAL, and root coverage). Their use is only recommended to improve early healing processes, especially in the donor bed, where they show good results.
6. In case PRF and L-PRF are used in periodontal plastic surgery procedures to perform root coverage with a CAF, it is recommended to use a minimum of three PRF or L-PRF membranes.
7. Greater protocol standardization is required to achieve an optimal effect of L-PRF in regenerative procedures, as well as in their clinical management.

## Figures and Tables

**Table 1:** Features of the reviews and meta-analyses evaluated.

Authors	Review type	Number of papers included	Type of periodontal regeneration	Types of platelet derivative	Main results
Miron et al. [17]	Systematic review	22	Intrabony defects and periodontal plastic surgery	PRF	Intrabony defect: additional use of PRF resulted in reduced PD and CAL gain compared to OFD (*P* < 0.05). Periodontal plastic surgery: CTG produced greater width in the keratinized tissue compared to PRF.
Miron et al. [18]	Systematic review and meta-analysis	27	Intrabony defects	PRF and L-PRF	Intrabony defect: higher overall CAL with PRF (0.84 (95% CI: 0.57–1.11) greater radiographic bone fill with PRF (0.99, 95% CI: 0.64–1.34).
Castro et al. [19]	Systematic review and meta-analysis	24	Intrabony defects and periodontal plastic surgery	PRF-L	Intrabony defect: PRF-L application led to reduced PD (mean difference: 1.1 mm, *P* < 0.001, CI: 0.6–1.6), and CAL gain (mean difference: 1, 2 mm, *P* < 0.001, CI: 0.5–1.9). Amount of bone fill in mm (mean difference: 1.7 mm, *P* < 0.001, CI: 1.0–2.3) and bone fill % (mean difference: 46.0%, 95% CI: 33.2–58.7). Periodontal plastic surgery: PRF-L produced a reduction in recession depth (mean difference: 0.6 mm, *P* < 0.01, CI: 0.2–1.1).
Panda et al. [20]	Systematic review and meta-analysis	15	Intrabony defects	PRF and PRP	Intrabony defect: bone fill %: PRF and PRP significantly higher than OFD alone. PRP + bone grafting caused significant improvement in CAL.
Zhou et al. [21]	Systematic review and meta-analysis	6	Intrabony defects	PRP and PRF	Intrabony defect: PD reduction: PRP and PRF presented statistically significant differences compared to DFDBA alone. PRP: mean deviation of (0.47; 95% CI: 0.14–0.80) and PRF: 0.88 (95% CI: 0.41–1.34) CAL gain: PRF statistically significant difference compared to DFDBA alone, mean deviation 0.77 (95% CI: 0.31–1.22). Greater with PRF than amniotic membrane. Bone fill %: PRP statistically significant difference compared to DFDBA alone; mean deviation 0.71 (95% CI: 0.13–1.29).
Moraschini and Barboza [24]	Systematic review and meta-analysis	7	Periodontal plastic surgery	PRF	Periodontal plastic surgery: improvement in healing and healing parameters. Postoperative comfort especially in the donor site.
Najeeb et al. [22]	Systematic review	13	Intrabony defects	PRF	Intraosseous defect: it was not reported a statistically significant additional benefit in clinical parameters with the use of PRF.
Tavelli et al. [23]	Systematic review and network meta-analysis	55	Intrabony defects	PRP, PRF, and L-PRF	Intraosseous defect: PD reduction: PRP (−0.41; 95% CI: −0.66 to − 0.16). PRF (−0.57; 95% CI: −0.76, −0.38) CAL gain: PRP (−0.58; 95% CI: −0.91 to − 0.26) PRF (−0.82; 95% CI: −1.08, −0.56). Bone fill %: PRP (17.32; 95% CI: 6.12–28.51). PRF (29.61; 95% CI: 23.28–35.93). EMD (19.71; 95% CI: 12.78, 26.64).

PD, probing depth; CAL, clinical attachment level; PRF, platelet-rich fibrin; PRP, platelet-rich plasma; L-PRF, leucocyte- and platelet-rich fibrin; OFD, surgical or open field therapy; DFDBA, demineralized bone matrix; CTG, connective tissue graft; EMD, emdogain.

**Table 2:** Primary studies included in the evaluated reviews.

Primary study	Study design	Follow-up in months	Outcome variables	Review including the primary study							
Authors				Miron et al. [17]	Miron et al. [18]	Castro et al. [19]	Panda et al. [20]	Zhou et al. [21]	Moraschini and Barboza [24]	Najeeb et al. [22]	Tavelli et al. [23]
Agarwal et al. [28]	Case control	6	Root coverage CAF versus CAF + AM versus CAF + PRF	X							
Agarwal et al. [29]	RCT	12	Intrabody defect regeneration OFD + DFDBA versus OFD + DFDBA + PRF	X	X	X		X		X	X
Agarwal et al. [30]	RCT	12	Intrabody defect regeneration OFD + DFDBA versus OFD + DFDBA + PRP					X			X
Ajwani et al. [31]	RCT	12	Intrabody defect regeneration OFD versus OFD + PRF-L	X	X	X				X	X
Aleksić et al. [32]	RCT	12	Root coverage CAF + CTG versus CAF + PRF	X							
Aroca et al. [33]	RCT	6	Root coverage CAF versus CAF + PRF	X		X			X		
Atchuta et al. [34]	RCT	6	Intrabody defect regeneration OFD versus OFD + ALLBG + PRF								X
Aydemir Turkal et al. [35]	RCT	6	Intrabody defect regeneration OFD + EMD versus OFD + PRF + EDM		X						
Bahammam and Attia [36]	RCT	6	Intrabody defect regeneration OFD + PRF versus OFD + SBG + PRF								X
Bajaj et al. [37]	RCT	9	Furcation degree II OFD versus OFD + PRF-L versus OFD + PRP			X					
Bansal and Bharti [38]	RCT	6	Intrabody defect regeneration OFD + DFDBA versus OFD + DFDBA + PRF		X	X		X		X	X
Bodhare et al. [39]	RCT	6	Intrabody defect regeneration OFD + bioguide versus OFD + bioguide +PRF		X						X
Camargo et al. [40]	RCT	9	Intrabody defect regeneration OFD + BPBM versus OFD + PRP + BPBM				X				
Chadwick et al. [41]	RCT	6	Intrabody defect regeneration OFD + DFDBA versus OFD + PRF		X						X
Chandradas et al. [42]	RCT	9	Intrabody defect regeneration OFD + PRF versus OFD + XBG + PRF								X
Chatterjee et al. [43]	RCT	9	Intrabody defect regeneration OFD + PRF versus OFD + TPRF (activated in titanium tubes)								X
Demir et al. [44]	RCT	9	Intrabody defect regeneration OFD versus OFD + PRP + SBG								X
Dogan et al. [45]	RCT	6	Root coverage CAF versus CAF + PRF	X							
Döri et al. [46]	RCT	12	Intrabody defect regeneration OFD versus OFD + PRF + HA				X				X
Elbehwashy et al. [47]	RCT	6	Intrabody defect regeneration OFD + PRF + ascorbic acid versus OFD + PRF								X
Elgendy and Abo Shady [48]	RCT	6	Intrabody defect regeneration OFD + HA nano bone® versus OFD + PRF-L + nano bone®	X	X	X				X	X
Eren and Atilla [49]	RCT	6	Root coverage CAF + CTG versus CAF + PRF	X		X			X		
Galav et al. [50]	RCT	9	Intrabody defect regeneration OFD + BAG versus OFD + PRF		X						X
Gamal et al. [51]	Case control	9	Intrabody defect regeneration OFD + XB versus OFD + PRGF versus OFD + PRF							X	X
Goyal et al. [52]	RCT	12	Intrabody defect regeneration ABBM + GTR + PRP versus ABBM + GTR				X				
Gupta et al. [53]	RCT	6	Intrabody defect regeneration OFD + EMD versus OFD + PRF		X	X				X	X
Gupta et al. [54]	RCT	6	Root coverage CAF versus CAF + PRF	X		X					
Hanna et al. [55]	RCT	12	Intrabody defect regeneration OFD + HA + sasline versus OFD + PRP + HA				X				X
Harnack et al. [56]	RCT	6	Intrabody defect regeneration OFD versus OFD + PRP								X
Hassan et al. [57]	RCT	12	Intrabody defect regeneration OFD versus OFD + ABG + PRP								X
Hazari et al. [58]	RCT	6	Intrabody defect regeneration OFD versus OFD + SBG + PRF								X
Ilgenli et al. [59]	RCT	6	Intrabody defect regeneration OFD + PRP versus OFD + ABG + PRP								X
Jalaluddin et al. [60]	RCT	6	Intrabody defect regeneration OFD versus OFD + PRP								X
Jalaluddin et al. [61]	RCT	6	Intrabody defect regeneration OFD versus OFD + PRP								X
Jankovic et al. [62]	RCT	9	Root coverage CAF + EMD versus CAF + PRF	X		X			X		
Jankovic et al. [63]	RCT	6	Root coverage CAF + CTG versus CAF + PRF	X		X			X		
Kang et al. [64]	Case control	6	Intrabody defect regeneration O FD versus OFD + PRP versus OFD + PRF				X				
Kanoriya et al. [65]	RCT	9	Intrabody defect regeneration OFD + PRF versus OFD + PRF + Alendronato 1%		X						X
Kaushick et al. [66]	RCT	6	Intrabody defect regeneration OFD versus OFD + SBG + PRP								X
Keceli et al. [67]	RCT	6	Root coverage CAF + CTG versus CAF + CGT + PRF	X		X					
Kukreja et al. [68]	RCT	6	Intrabody defect regeneration OFD + DFDBA versus OFD + DFDBA + PRP					X			X
Lekovic et al. [69]	RCT	6	Intrabody defect regeneration OFD + PRF-L versus OFD + PRF-L + BMPM			X	X			X	X
Markou et al. [70]	RCT	6	Intrabody defect regeneration OFD versus OFD + PRP + ABG								X
Martande et al. [71]	RCT	9	Intrabody defect regeneration OFD versus OFD + PRF versus OFD + PRF + Atorvastatina 1.2%		X						X
Mathur et al. [72]	RCT	6	Intrabody defect regeneration OFD + ABG versus OFD + PRF		X	X				X	X
Menezes et al. [73]	RCT	12	Intrabody defect regeneration OFD + DFDBA + PRP versus OFD + DFDBA				X				
Naqvi et al. [74]	RCT	9	Intrabody defect regeneration OFD versus OFD + BAG versus OFD + PRF + BAG		X						X
Okuda et al. [75]	Case control	12	Intrabody defect regeneration OFD versus OFD + PRP + SBG								X
Ozdemir and Okte [76]	RCT	6	Intrabody defect regeneration OFD + ABBM versus OFD + PRP + ABBM				X				X
Padma et al. [77]	RCT	6	Root coverage CAF versus CAF + PRF	X		X			X		
Panda et al. [78]	RCT	9	Intrabody defect regeneration OFD + barrier membrane versus OFD + barrier membrane + PRF	X							
Paolantonio et al. [79]	RCT	12	Intrabody defect regeneration OFD versus OFD + PRF-L + ABG								X
Patel et al. [80]	RCT	12	Intrabody defect regeneration OFD versus OFD + PRF								X
Pavani et al. [81]	RCT	6	Intrabody defect regeneration OFD versus OFD + PRF + SBG								X
Pham [82]	RCT	12	Intrabody defect regeneration OFD + PRF versus GRT versus OFD								X
Pradeep et al. [83]	Case control	9	Effects on intrabody regeneration OFD versus OFD + PRP				X				X
Pradeep et al. [84]	RCT	9	Intrabody defect regeneration OFD versus OFD + PRP versus OFD + PRF-L	X	X	X	X			X	X
Pradeep et al. [85]	RCT	9	Intrabody defect regeneration OFD + 1% MF versus OFD + PRF versus OFD + 1% MF + PRF	X	X	X				X	X
Pradeep et al. [86]	RCT	9	Intrabody defect regeneration OFD versus OFD + PRF + SBG								X
Pradeep et al. [87]	RCT	9	Intrabody defect regeneration OFD versus OFD + PRF versus OFD + PRF + Rosuvastatina 1.2%		X						X
Rajaram et al. [88]	RCT	24	Root coverage DLSBF versus DLSBF + PRF	X							
Rosamma et al. [89]	RCT	12	Intrabody defect regeneration OFD versus OFD + PRF-L		X	X					
Sezgin et al. [90]	RCT	6	Intrabody defect regeneration OFD versus ABBM + PRP versus OFD + ABBM		X						X
Shah et al. [91]	RCT	6	Intrabody defect regeneration OFD + DFDBA versus OFD + PRF-L	X	X	X				X	X
Sharma and Pradeep [92]	RCT	9	Intrabody defect regeneration OFD versus OFD + PRF	X	X	X	X				
Sharma and Pradeep [93]	RCT	9	Furcation degree II OFD + PRF versus OFD + PRF-L	X		X				X	X
Shukla et al. [94]	RCT	9	Intrabody defect regeneration OFD versus OFD + SBG + PRP								X
Thamaraiselvan et al. [95]	Case control	6	Root coverage CAF versus CAF + PRF	X		X			X		
Thorat et al. [96]	RCT	9	Intrabody defect regeneration OFD versus OFD + PRF	X	X	X	X			X	X
Tunalɩ et al. [97]	RCT	12	Root coverage CAF + CTG versus CAF + PRF	X		X			X		
Yajamanya et al. [98]	RCT	9	Intrabody defect regeneration OFD + BAG versus OFD + PRF		X						X
Yilmaz et al. [99]	RCT	12	Intrabody defect regeneration GTR + BM + PRP versus BM + GTR				X				

CAF, coronary displacement flap; EMD, Emdogain; CTG, connective tissue graft; DLSBF, double lateral sliding bridge flap; AM, amniotic membrane; OFD, surgical therapy; DFDBA, demineralized bone matrix; PRP, platelet-rich plasma; L-PRF, leucocyte-and platelet-rich fibrin; MF, metformin; HA, hydroxyapatite; BM, barrier membrane; ABG, autogenous bone graft; BAG, bioactive glass; ABBM, bovine anorganic graft; BMPM, bone morphogenic protein; SBG, synthetic bone graft; PRGF, platelet-derived growth factor; XB, bovine xenograft; BM, bone mineral; ALLBG, allogenic bone graft; RCT, randomized clinical trial.

**Table 3:** Quality of the selected studies.

Authors	PRISMA quality of report	Risk of bias	AMSTAR-2 confidence level	Scale used to evaluate primary studies
Miron et al. [17]	18/27	Moderate	Low	Not reported
Miron et al. [18]	25/27	Low	Low	Cochrane risk-of-bias tool for randomized trials
Castro et al. [19]	23/27	Low	Low	Cochrane collaboration's tool for assessing the risk of bias
Panda et al. [20]	23/27	Low	Low	Cochrane
Zhou et al. [21]	23/27	Low	Low	Cochrane
Moraschini and Barboza [24]	27/27	Low	High	Cochrane collaboration's tool
Najeeb et al. [22]	18/27	Moderate	Low	Jadad scale
Tavelli et al. [23]	23/27	Low	High	Cochrane risk-of-bias tool for randomized trials

## Data Availability

Records were obtained from the included investigations.
